# MicroRNA‐425‐5p regulates chemoresistance in colorectal cancer cells *via* regulation of Programmed Cell Death 10

**DOI:** 10.1111/jcmm.12742

**Published:** 2015-12-09

**Authors:** Ye Zhang, Xingqian Hu, Xiaofei Miao, Kuiyu Zhu, Songkui Cui, Qingyang Meng, Jialin Sun, Tong Wang

**Affiliations:** ^1^ Wuxi People's HospitalWuxiJiangsuChina; ^2^ Nanjing Medical UniversityNanjingJiangsuChina

**Keywords:** colorectal cancer, miR‐425‐5p, chemoresistance, PDCD10

## Abstract

Acquired chemoresistance represents a major obstacle in cancer treatment, the underlying mechanism of which is complex and not well understood. MiR‐425‐5p has been reported to be implicated tumorigenesis in a few cancer types. However, its role in regulating chemoresistance has not been investigated in colorectal cancer (CRC) cells. Microarray analysis was performed in isogenic chemosensitive and chemoresistant HCT116 cell lines to identify differentially expressed miRNAs. miRNA quantitative real‐time PCR was used to detect miR‐425‐5p expression levels between drug resistant and parental cancer cells. MiR‐425‐5p mimic and inhibitor were transfected, followed by CellTiter‐Glo^®^ assay to examine drug sensitivity in these two cell lines. Western Blot and luciferase assay were performed to investigate the direct target of miR‐425‐5p. Xenograft mouse models were used to examine *in vivo* function of miR‐425‐5p. Our data showed that expression of miR‐425‐5p was significantly up‐regulated in HCT116‐R compared with parental HCT116 cells. Inhibition of miR‐425‐5p reversed chemoresistance in HCT116‐R cells. Programmed cell death 10 (PDCD10) is the direct target of miR‐425‐5p which is required for the regulatory role of miR‐425‐5p in chemoresistance. MiR‐425‐5p inhibitor sensitized HCT116‐R xenografts to chemo drugs *in vivo*. Our study demonstrated that miR‐425‐5p regulates chemoresistance of CRC cells by modulating PDCD10 expression level both *in vitro* and *in vivo*. MiR‐425‐5p may represent a new therapeutic target for the intervention of CRC.

## Introduction

Colorectal cancer (CRC) is the third most commonly diagnosed cancer and third leading cause of cancer‐related mortality in the United States with 133,000 estimated new cases and 50,310 estimated deaths in 2015 (American Cancer Society). In the last decade, the use of chemotherapeutic agents in monotherapy or in combined regimens has markedly increased the survival of patients with CRC at stages III and IV. Unfortunately, intrinsic or acquired drug resistance of cancer cells continues to impose a great obstacle to survival in patients with metastatic chemoresistant CRC 1, 2. Previous studies have indicated that the mechanisms underlying chemoresistance of cancer cells is complex, involving multiple processes, such as drug efflux, autocrine survival signalling, and alterations in DNA synthesis and damage repair, cell survival and apoptosis, *etc*. 3, 4. More recent studies have shown that both genetic changes including mutations, translocations, deletions and amplification of genes or promoter regions and epigenetic changes including aberrant DNA methylation, histone modifications, and non‐coding RNA expression may also contribute to acquired drug resistance of cancer cells 5, 6.

MicroRNAs (miRNAs) are a class of endogenous small non‐coding RNAs of 18–24 nucleotides that post‐transcriptionally regulate genes expression 7. MiRNAs play crucial roles in diverse biological processes, such as cell apoptosis, cell proliferation, stress response and metabolism 8. In recent years, a growing body of evidence has demonstrated that many miRNAs are dysregulated in many cancers, such as breast, prostate, colon and lung 9, 10. miRNAs can function as onco‐miRs or tumour‐suppressor miRs depending on their target genes 11. Previous studies have also shown that miRNAs are involved in modulating the sensitivity of cancer cells to chemotherapeutic drugs and dysregulation of miRNA function might contribute to the acquisition of chemoresistance 12, 13. For example, miR‐21 has been found to be up‐regulated in various chemoresistant cancer cells and down‐regulation of miR‐21 *via* anti‐miR‐21 oligonucleotide sensitized those resistant cells to apoptosis 14, 15, 16; overexpression of miR‐181s *via* transfection of miR‐181s mimics led to increased CDDP‐induced apoptosis in a multidrug‐resistant human lung cancer cell line A549/CDDP (cisplatin) 16; miR‐200c has been reported to regulate chemoresistance in several cancer cells through different mechanisms 17, 18, 19. Most recently, miR‐425‐5p has been reported to be implicated in tumorigenesis in many cancer types 20, 21, 22, 23. However, the role of miR‐425‐5p in regulating chemoresistance and the underlying mechanism have not been investigated in CRC cells.

In this study, we examined the involvement of miR‐425‐5p in regulating chemoresistance to 5‐fluorouracil (5‐FU) and oxaliplatin (OX) in CRC cells using two isogenic HCT116 cell lines which is sensitive or resistant to these two agents. We provided evidence that miR‐425‐5p can directly modulate chemoresistance in these cells by regulating the expression level of its downstream target PDCD10 both *in vitro* and *in vivo*.

## Materials and methods

### Cell culture

Human colon cancer cell line HCT116 was purchased from ATCC and was cultured in basal medium supplemented with 10% serum at 37°C and 5% CO_2_. HCT116‐R cell line was generated by incubating HCT116 cells with increasing level of 5‐FU (Sigma‐Aldrich, St. Louis, MO, USA) together with OX (Sigma‐Aldrich) until the concentration reached 2 μM for OX and 2 μg/ml for 5‐FU. Resistant cell lines were maintained under constant treatment with drug for daily culture.

### Chemo‐sensitivity assay

Cells were seeded at a density of 5 × 10^3^ cells/well in 96‐well microtitre plates and allowed to attach overnight. 5‐FU or OX alone was then added and cultured for an additional 72 hrs. Cell viability was assessed using CellTiter‐Glo^®^ assay. Each value was normalized to cells treated with DMSO and the IC_50_ values are calculated using Graphpad Prism software. Each assay was performed in biological duplicates.

### Microarray analyses

The hybridized Human Genome U133A 2.0 Array (Affymetrix, Santa Clara, CA, USA) was scanned and analysed with the Affymetrix Microarray Analysis Suite version 5.0. The average density of hybridization signals from three independent samples was used for data analysis, and genes with signal density less than 300 pixels were omitted from the analysis. The *P*‐values were calculated with two‐sided *t*‐tests with unequal variance assumptions. To correct for multiple hypothesis testing, the false discovery rate was calculated. Differentially expressed genes were selected using both a false discovery rate of less than 0.01 and a fold‐change greater than 1.5 or less than 21.5.

### Plasmids and cell transfection

Cells in logarithmic growth phase were prepared for cell transfection. Transfection of miR‐425‐5p inhibitor, miR‐425‐5p mimic and its non‐specific control (Invitrogen, Hampton, VA, USA) were performed according to the manual provided with the siPORTM NeoFXTM Transfection Agent (Ambion, Grand Island, NE, USA). pLenti‐C‐Myc‐DDK PDCD10 cDNA (RC221815L1) and two individual siRNAs (SR307707) targeting PDCD10 were obtained from Origene. siRNAs were transfected with Lipofectamine RNAi Max reagent (Invitrogen, Grand Island, NE, USA) as per manufacturer's protocol. cDNA transfections were performed with Lipofectamine LTX reagent (Invitrogen) as per manufacturer's protocol.

### Real‐time PCR

The cells or spheroids were harvested after the transfection and the RNA was isolated using TRI reagent (Sigma‐Aldrich). Ten ng RNA was used for reverse transcription using the TaqMan MicroRNA RT Kit (Applied Biosystems, Life Technologies, Madison, WI, USA). Briefly, 5 μl of the RNA was added to 10 μl of the master mix containing 0.15 μl dNTP (100 nM), 1 μl multiscribe enzyme (50 U/μl), 1.5 μl 10× RT‐puffer, 0.19 μl RNAse inhibitor (20 U/μl), 4.16 μl RNAse free H_2_O and 3 μl of primers (miR‐200a/b/c, miR‐141). The conditions for the reverse transcription were 30 min. at 16°C, 30 min. at 42°C and 5 min. at 85°C. Quantitative real‐time PCR of the individual miRNAs was performed in a total volume of 10 μl containing 5 μl TaqMan master mix, 3.17 μl RNase free H_2_O, 0.5 μl TaqMan primer and 1.33 μl cDNA. The PCR was performed in quadruple in a Rotor‐Gene Corbett 6000 Q PCR. The conditions for the reaction were: 2 min. at 50°C, 10 min. at 95°C, cycling (50 repeats): step 1 15 sec. at 95°C, step 2 60 sec. at 60°C. The data were collected and analysed using the quantitative Rotor‐Gene software. The miRNA levels were normalized to the stable internal control miRNA RNU 6B using the algorithm of Pfaffl. The RT‐PCR primers (cat no. 204337) were purchased from Exiqon (Woburn, MA, USA).

### Caspase 3/7 activity

Control or miR‐425‐5p transfected cells were treated with vehicle control or 5‐FU or OX for 72 hrs and subjected to Caspase‐Glo 3/7 assay according to the manufacturer's instructions in 96‐well plates (Promega, Madison, WI, USA).

### Immunoblot analysis

Total cell lysates were prepared by harvesting cells in Laemmli S.D.S reducing buffer [50 mM Tris‐HCl (pH 6.8), 2% S.D.S, and 10% glycerol], boiled and resolved on an 8–10% polyacrylamide gel, and transferred to polyvinylidinefluoride. Antibodies against PDCD10 (Abcam, Cambridge, MA, USA), and β‐actin (Sigma‐Aldrich) were used. The blots were incubated with horseradish peroxidase‐conjugated donkey anti‐rabbit or antimouse IgG (Santa Cruz Biotechnology, Dallas, TX, USA) at a dilution of 1:5000 and detected with SuperSignalWest Pico or Femto Chemiluminescent Substrate Kit (Thermo Scientific, Grand Island, NE, USA).

### Luciferase assay

Two hundred and fifty nanograms of pGL3 reporter vector carrying the WT or mutant miR‐425‐5p‐binding site (see plasmid construct, Fig. 4D), 25 ng of the phRL‐SV40 control vector (Promega), and 100 nM miRNA precursors or scrambled sequence miRNA control (Ambion) were cotransfected into HEK293 cells in 24‐well plates. Firefly luciferase activity was measured with a Dual Luciferase Assay Kit (Promega) 24 hrs after transfection and normalized with a Renilla luciferase reference plasmid.

### Immunohistochemistry staining

The paraffin‐embedded sections were subjected to antigen retrieval by heating the slides in a microwave at 100°C for 10 min. in 0.1 M citric acid buffer (pH = 6.0), and then incubated with corresponding antibodies at 4°C overnight. After secondary antibody incubation at room temperature for 1 hr, the slides were developed in 0.05% diaminobenzidine containing 0.01% hydrogen peroxidase. For negative controls, specific antibodies were replaced with normal goat serum by co‐incubation at 4°C overnight preceding the immunohistochemical staining procedure.

### Xenograft experiments

All animal experiments were approved by Institutional Animal Care and Use Committee of National Cancer Center. HCT116‐R cells (3 × 10^6^ cells/injection) were subcutaneously injected into both flanks of 5 weeks old female nude mice group. Vehicle, miR‐425‐5p inhibitor, 5‐FU (25 mg/kg), OX (25 mg/kg), alone or combined were injected i.p. into mice daily for 12 days. Tumour volumes were measured using calliper and determined by a formula [volume = (length × width2)/2] from day 6 to day 18 post implantation. The results were expressed as mean tumour volumes with SD. The protocol was approved by the Committee on the Ethics of Animal Experiments of Nanjing Medical University. All surgery was performed under sodium pentobarbital anaesthesia by i.p. injection at a concentration of 100 mg/kg, and all efforts were made to minimize suffering.

### Statistical analysis

Quantitative data are expressed as mean ± S.D. Statistical significance was assessed by the Student's *t*‐test when there are only two groups. For other situations, one‐way anova followed by *post hoc* test is performed. Differences were considered to be significant when *P* < 0.05.

## Results

### MiR‐425‐5p is up‐regulated in chemo‐resistant HCT116 cells compared to its isogenic parental cells

**Figure 1:**
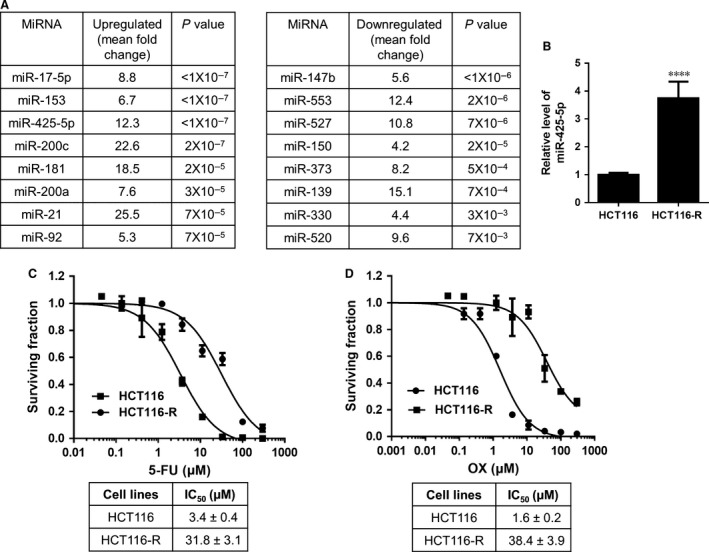
Characterization of chemosensitivity and miR‐425‐5p expression in HCT116 and HCT116‐R cells. (**A**) miRNA expression profiles were determined in HCT116 and HCT116‐R cells. Top 8 up‐regulated and down‐regulated miRNAs ranked by *P*‐value in HCT116‐R cell line compared with HCT116 were listed in the table. (**B**) Validation of down‐regulation of miR‐425‐5p in HCT116‐R cells compared with HCT116 parental cells. The relative level of miR‐425‐5p was determined using qRT‐PCR and normalized to parental HCT116 cells. (**C** and **D**) Dose–response curves of HCT116 and HCT116‐R cell lines towards 5‐fluorouracil (5‐FU) and oxaliplatin (OX): 5‐FU (**C**) and OX (**D**). IC50 values were listed in the tables below. Cells were seeded at a density of 5 × 10^3^ cells/well in 96‐well microtitre plates and allowed to attach overnight. 5‐FU or L‐OX alone was then added and cultured for an additional 72 hrs. Cell viability was assessed using CTG assay. Data represent the mean ± S.D.,* n* = 3. ****P < 0.0001,

We generated isogenic chemoresistant HCT116 cells (HCT116‐R) by incubating HCT116 cells with increasing concentration of 5‐FU and OX continuously until the concentration reached clinically relevant levels. The combination of these two chemo drugs is a common chemotherapy regimen for CRC patients in clinic. Chemosensitivity assays showed that the derived HCT116‐R cells were more resistant towards 5‐FU and OX compared to the parental HCT116, with about 10‐ and 20‐fold increase in IC50 values respectively (Fig. 1C and D). Longer period of incubation with these two drugs did not change their sensitivity profiles (data not shown). Microarray analysis was performed in these two cell lines to identify miRNAs involved in regulating chemoresistance in these cells. The top 8 miRNAs that are most significantly up‐ or down‐regulated in HCT116‐R cells were listed in Figure 1A. Among the top 3 ranked up‐regulated miRNAs, miR‐425‐5p exhibited the greatest fold changes. We measured the endogenous level of miR‐425‐5p in these two cell lines using real‐time PCR and confirmed that miR‐425‐5p was significantly up‐regulated in HCT116‐R cells (Fig. 1B).

### Inhibition of miR‐425‐5p sensitized HCT116‐R cells to 5‐FU and OX with concomitant increased induction of apoptotic cell death

**Figure 2:**
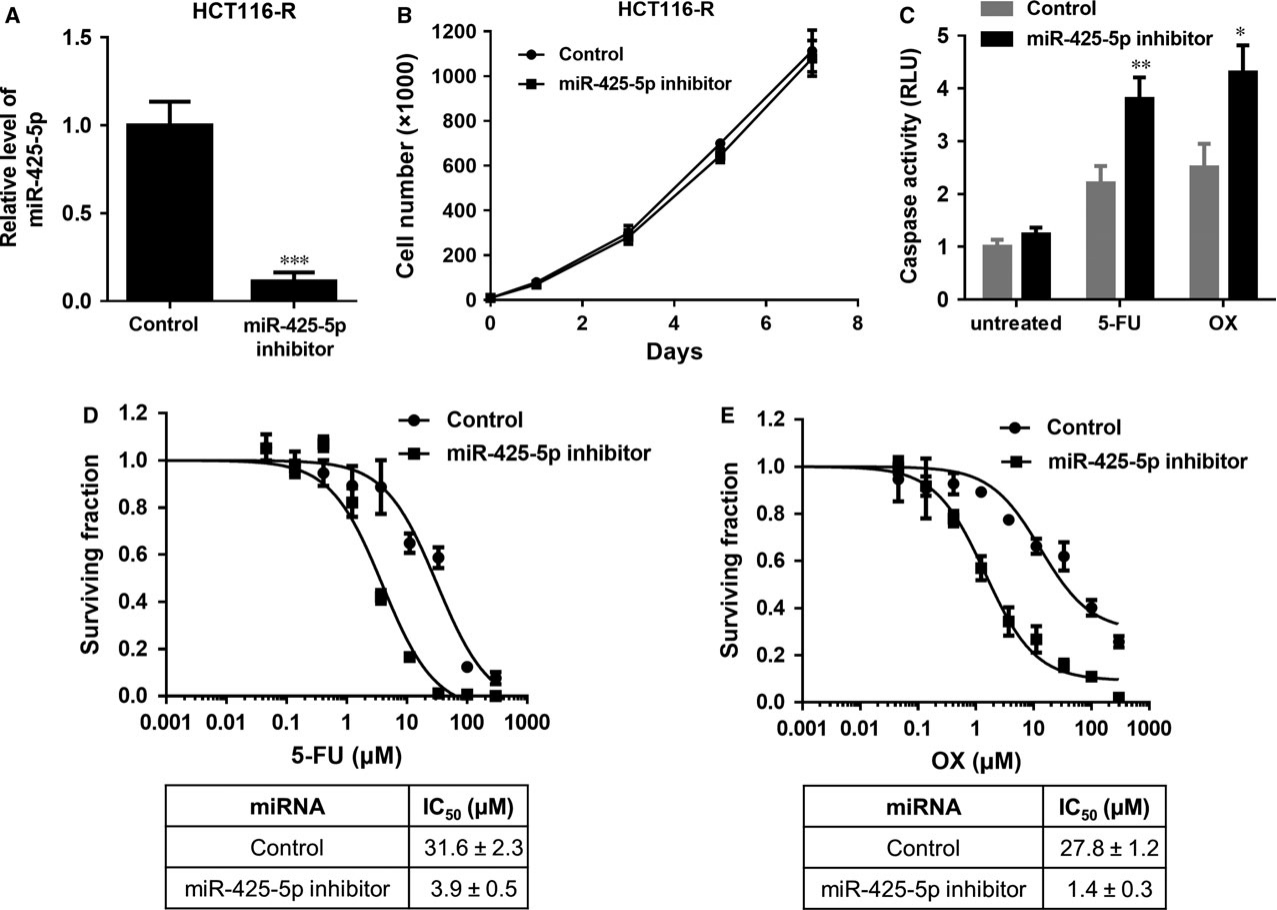
Inhibition of miR‐425‐5p sensitized HCT116‐R cells to 5‐fluorouracil (5‐FU) and oxaliplatin (OX) in HCT116 cells. (**A**) Relative level of miR‐425‐5p in HCT116‐R cells transfected with control miRNA or miR‐425‐5p inhibitor. (**B**) Growth curves of HCT116‐R cells transfected with control or miR‐425‐5p inhibitor. (**C**) Caspase activity in control and miR‐425‐5p inhibitor transfected cells treated with or without 5‐FU and OX. (**D** and **E**) Chemosensitivity of HCT116‐R cells transfected with control miRNA or miR‐425‐5p inhibitor to 5‐fluorouracil (5‐FU) and oxaliplatin (OX): 5‐FU (**D**) and OX (**E**). IC50 values were listed in the tables below. Dose–response studies were performed as described in Figure 1. Data represent the mean ± S.D.,* n* = 3. *P < 0.05, **P < 0.01, ***P < 0.001.

To investigate the role of miR‐425‐5p in chemo‐resistance in CRC cells, miR‐425‐5p inhibitor was transfected into HCT116‐R cells. Transfection of HCT116 with miR‐425‐5p inhibitor markedly reduced miR‐425‐5p level as shown in Figure 2A, which did not affect cell proliferation (Fig. 2B). The cells transfected with miR‐425‐5p inhibitor were much more sensitive to both 5‐FU and OX (Fig. 2D and E) with about 8‐ and 20‐fold decrease in IC50 respectively. Correspondingly, inhibition of miR‐425‐5p induced more caspase 3/7 activity compared to control cells (Fig. 2C), suggesting that miR‐425‐5p is a key mediator for chemoresistance in these cells.

### Overexpression of miR‐425 confers resistance to 5‐FU and OX in HCT116 cells

**Figure 3:**
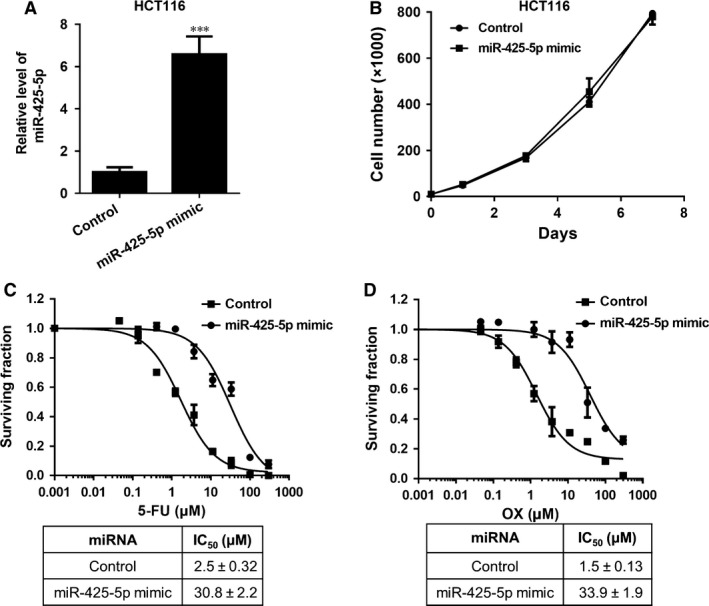
Overexpression of miR‐425‐5p confers resistance of HCT116 cells to 5‐fluorouracil (5‐FU) and oxaliplatin (OX). (**A**) Relative level of miR‐425‐5p in HCT116 cells transfected with control miRNA or miR‐425‐5p mimic. Data represent mean ± S.D. ***P < 0.001. (**B**) Growth curves of HCT116 cells transfected with control or miR‐425‐5p mimic. (**C** and **D**) Chemosensitivity of HCT116 cells transfected with control miRNA or miR‐425‐5p mimic to 5‐fluorouracil (5‐FU) and oxaliplatin (OX): 5‐FU (**C**) and OX (**D**). IC50 values were listed in the tables below. Dose–response studies were performed as described in Figure 1. Data represent the mean ± S.D.,* n* = 3.

To further confirm the role of miR‐425‐5p in regulating chemoresistance, we overexpressed this miRNA in HCT116 cells and performed chemosensitivity assay. The cells transfected with miR‐425‐5p mimic exhibited ~6‐fold higher level of miR‐425‐5p compared to the cells transfected with non‐specific negative control (Fig. 3A). These miR‐425‐5p overexpression cells showed no difference in cell proliferation rate (Fig. 3B) and they were much more resistant to 5‐FU and OX than the control cells (Fig. 3C and D).

### MiR‐425‐5p directly targets the 3′UTR of PDCD10 in colon cancer cells

**Figure 4:**
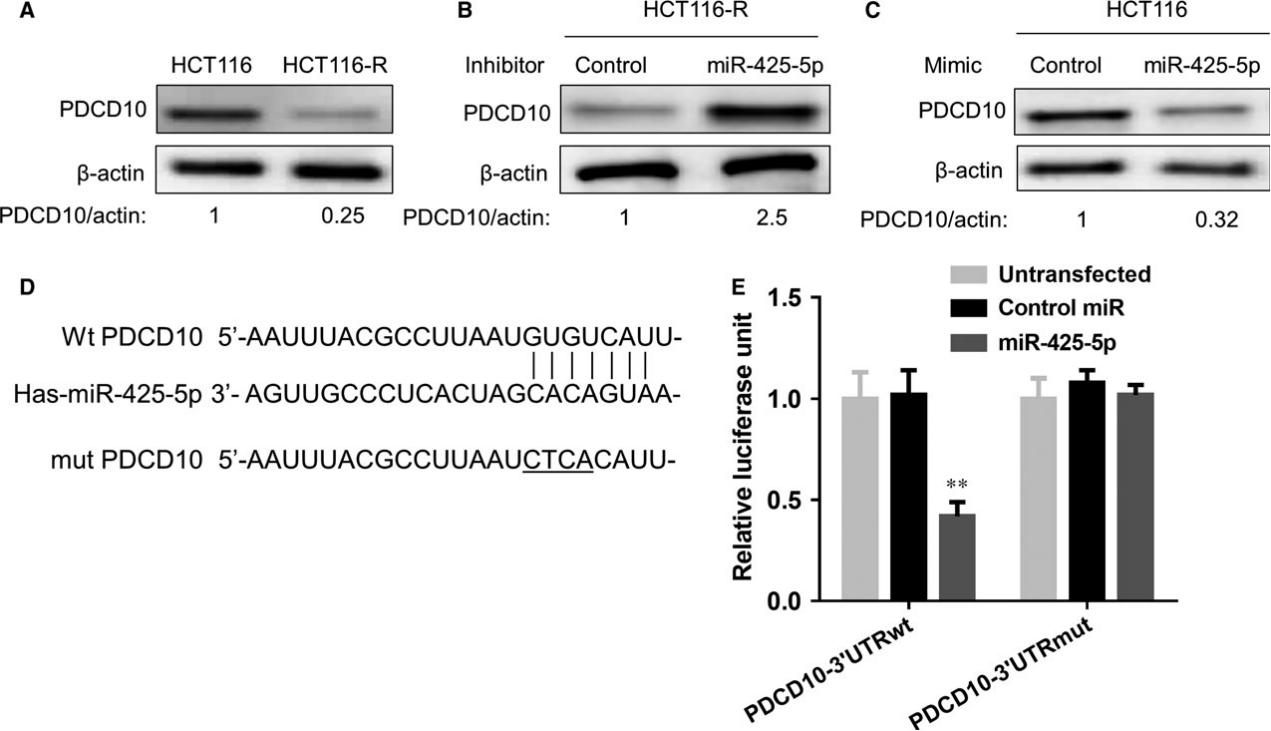
miR‐425‐5p directly targets the 3′UTR of PDCD10 in colon cancer cells. (**A**) Western Blot analysis of PDCD10 protein level in HCT116 and HCT116‐R cells. β‐actin was used as loading control. (**B**) Western Blot analysis of PDCD10 protein level in HCT116‐R cells transfected with control or miR‐425‐5p inhibitor. β‐actin was used as loading control. (**C**) Western Blot analysis of PDCD10 protein level in HCT116 cells transfected with control or miR‐425‐5p mimic. β‐actin was used as loading control. (**D**) The wild‐type 3′‐UTR of mammalian PDCD10 mRNA contains a putative miR‐425‐5p‐binding site. The mutant form was shown below. (**E**) Luciferase reporter assays. Cells were transfected with reporters containing the wild‐type or mutant form after transfection with miR‐425‐5p mimics or control miRNA. Data represent the mean ± S.D.,* n* = 3. **P < 0.01.

To explore potential gene targets of miR‐425‐5p that may be involved in this effect, we examined for putative targets using miRNA target prediction programmes, miRanda (www.microrna.org), TargetScan (www.targetscan.org) and Diana‐microTv3.0 (http://diana.cslab.ece.ntua.gr/microT/). Among all the candidate genes, PDCD10 showed up in two of these three programmes. This protein has been implicated in regulating apoptosis 24. Western blot analysis showed that there was a down‐regulation of PDCD10 proteins in HCT116‐R cells (Fig. 4A), which correlated with miR‐425‐5p level. Inhibition of miR‐425‐5p led to increased protein level of PDCD10 in HCT116‐R cells (Fig. 4B), whereas overexpression of miR‐425‐5p reduced PDCD10 level in HCT116 cells (Fig. 4C). To further validate PDCD10 is the direct target of miR‐425‐5p, we cloned the 3′‐UTR of PDCD10 containing the single putative miR‐425‐5p‐binding site downstream of the Renilla luciferase open reading frame. Both wild‐type 3′‐UTR and a mutant form in which the putative seed‐binding site was mutated were evaluated using luciferase assay (Fig. 4D). As shown in Figure 4E, cotransfection of miR‐425‐5p precursor with wild‐type PDCD103′‐UTR reporter construct significantly repressed relative luciferase activity, whereas mutation of the miR‐425‐5p‐binding site eliminated this effect, supporting that PDCD10 is a direct target of miR‐425‐5p in CRC cells.

### MiR‐425‐5p regulates chemoresistance through PDCD10

**Figure 5:**
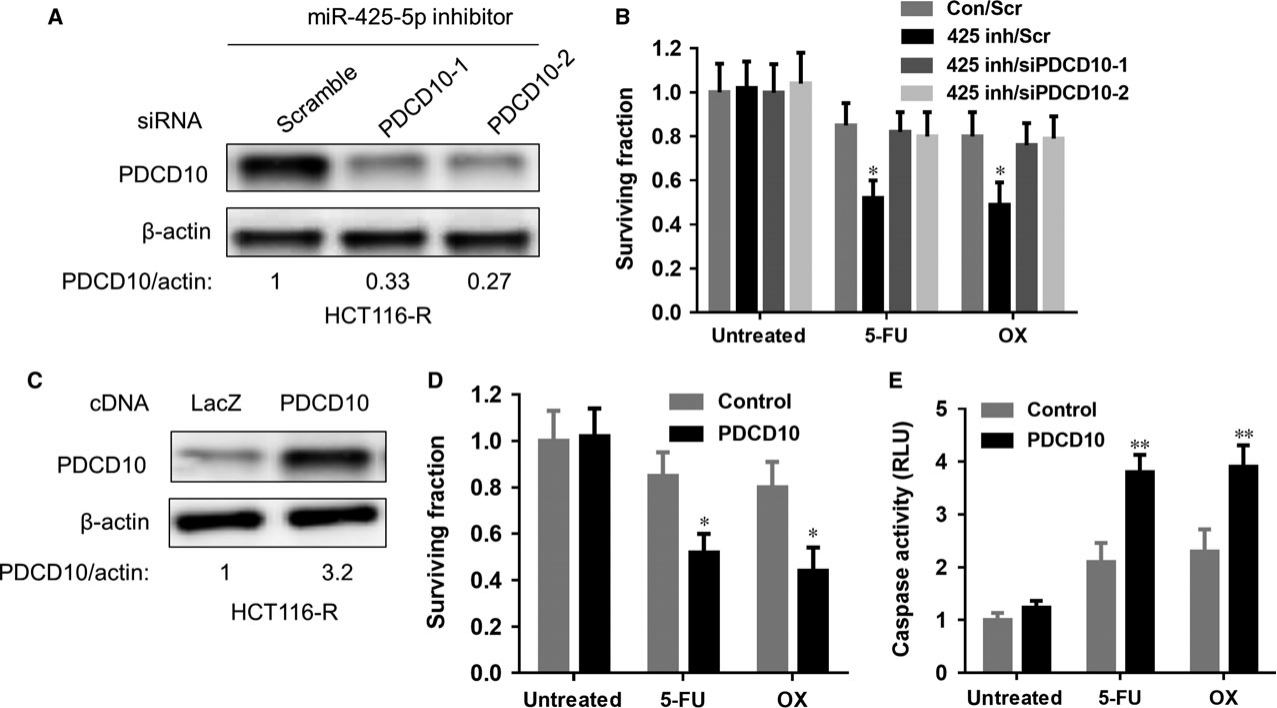
miR‐425‐5p regulates chemoresistance through PDCD10. (**A**) Western blot analysis of PDCD10 protein level in HCT116‐R cells cotransfected with miR‐425‐5p inhibitor and scramble or PDCD10 siRNAs. β‐actin was used as loading control. (**B**) Western Blot analysis of PDCD10 protein level in HCT116‐R cells transfected with PDCD10 cDNA. β‐actin was used as loading control. (**C**) Transient knockdown of PDCD10 reversed the chemosensitizing effects of miR‐425‐5p inhibitor in HCT116‐R cells with 5‐FU (5 μM) or OX (10 μM) treatments. Data represent the mean ± S.D.,* n* = 2. *P < 0.05. 425 inh: miR‐425‐5p inhibitor; Scr: scramble miRNA; (**D**) Overexpression of PDCD10 phenocopied the effects of miR‐425‐5p inhibitor to sensitize HCT116‐R to 5‐FU (5 μM) or OX (10 μM). Data represent mean ± S.D.,* n* = 3. *P < 0.05. (**E**) Caspase activity in control and PDCD10 overexpression cells treated with or without 5‐FU and OX. Data represent mean ± S.D.,* n* = 3. **P < 0.01.

To determine the importance of PDCD10 in the regulatory role of miR‐425‐5p in drug resistance, we transiently knocked down PDCD10 in HCT116‐R cells transfected with miR‐425‐5p inhibitor (Fig. 5A). Although inhibition of miR‐425‐5p reversed the drug resistance, knockdown of PDCD10 eliminated this sensitizing effect (Fig. 5C). Additionally, ectopic expression of a recombinant PDCD10 in HCT116‐R cells phenocopied the effect of miR‐425‐5p inhibition by sensitizing these cells to 5‐FU and OX (Fig. 5B and D). These results suggest that PDCD10 is required for miR‐425‐5p's functions in regulating chemoresistance.

### MiR‐425‐5p regulates chemoresistance of colon tumours *in vivo*

**Figure 6:**
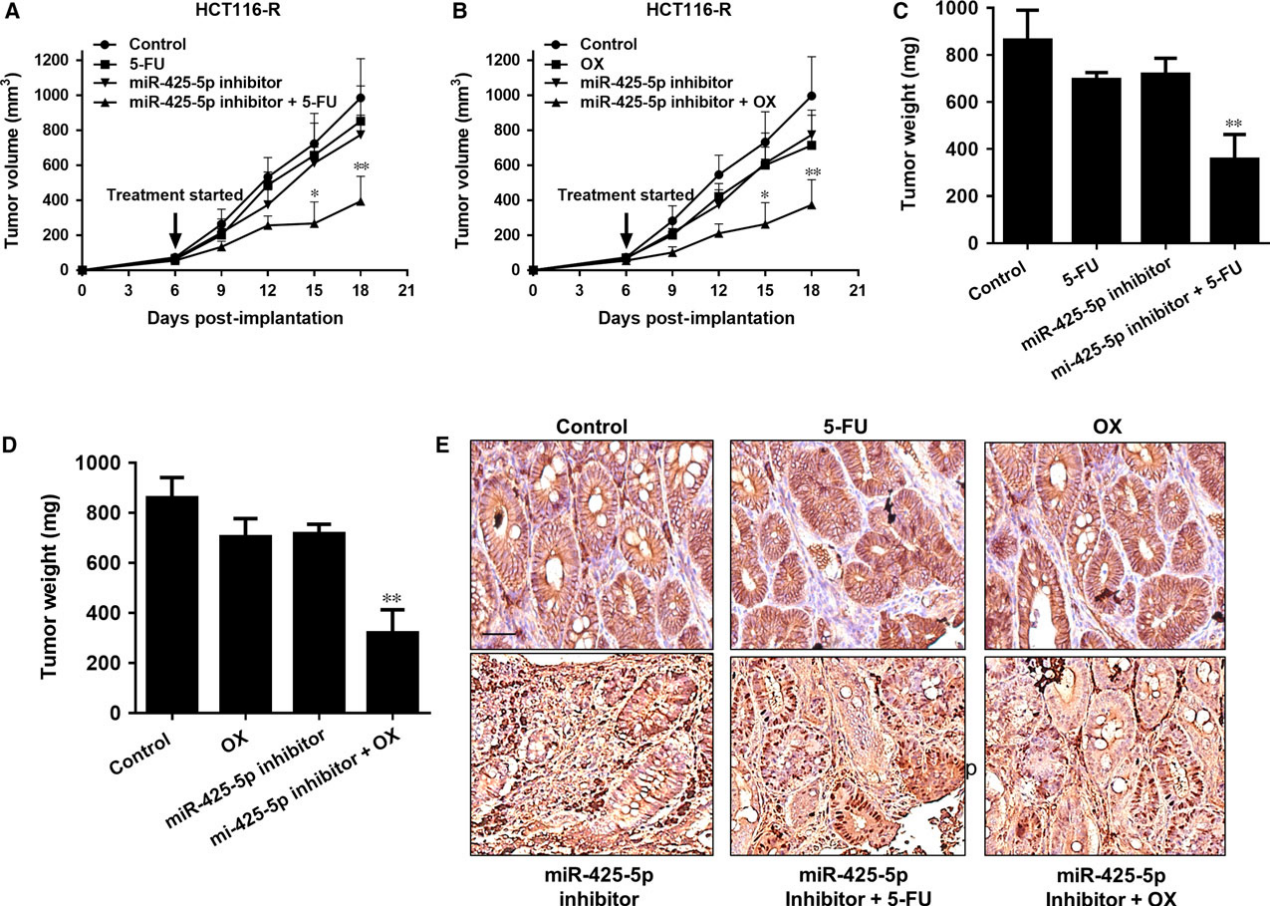
miR‐425‐5p regulates chemoresistance of colon tumours *in vivo*. (**A** and **B**) Co‐administration of miR‐425‐5p inhibitor enhanced tumour growth inhibitory effects of 5‐FU and OX *in vivo*. HCT116 cells were subcutaneously implanted into nude mice and received i.p. injection of either miR‐425‐5p inhibitor, 5‐FU (25 mg/kg) or OX (25 mg/kg) lone or combined. Tumour volumes (mean ± S.D.) were obtained from five mice for each group and plotted. Data represent mean ± S.D. *P < 0.05, **P < 0.01 *versus* the vector control. (**C** and **D**) Tumour weights of the mice treated as described above. Data represent mean ± S.D. **P < 0.01. (**E**) Representative IHC staining of PDCD10 in control and miR‐425‐5p inhibitor‐transfected xenografts. The scale bar indicates 50 μm.

To validate the regulatory role of miR‐425‐5p in chemoresistance *in vivo*, we examined the effects of miR‐425‐5p inhibition on chemoresistance of HCT116‐R cells using xenograft mouse model. As shown in Figure 6A and B, administration of 5‐FU or OX alone did not inhibit tumour growth in these mice, but did significantly inhibit tumour growth and reduce tumour weight when miR‐425‐5p inhibitor was co‐administered (Fig. 6A–D). Immunohistochemistry (IHC) analysis showed that the xenografts derived from mice administered with miR‐425‐5p inhibitor showed reduced expression of PDCD10 compared to the control xenografts (Fig. 6E). Taken together, these results suggest that miR‐425‐5p can regulate chemoresistance and its downstream target PDCD10 *in vivo*.

## Discussion

5‐fluorouracil and OX are standard therapy for metastatic CRC. Their use in combination with leucovorin (FOLFOX) for metastatic CRC has led to response rates >50% and median survival approaching 2 years 25. In spite of the fact that recent therapeutic regimens have significantly increased survival in metastatic disease, development of chemoresistance is inevitable. Therefore, it is essential to understand the mechanisms of resistance in CRC to optimize current therapeutic strategies. To investigate the potential mechanism of chemoresistance in CRC cells, we generated a chemoresistant cell line from the parental human CRC cell line HCT116. HCT116‐R cells were developed to be resistant to 5‐FU and OX, respectively, at the clinically relevant plasma concentrations of patients receiving these drugs (Fig. 1C and D) 26.

Recently, a growing body of evidence has suggested that miRNAs may play important roles in chemotherapy resistance in many cancer types 27. For example, miR‐15b, miR‐16 and miR‐497 were found to be down‐regulated in multidrug‐resistant gastric cancer cell line SGC7901/VCR (vincristine) and overexpression of these miRNAs could sensitize cells to anti‐cancer drugs by targeting anti‐apoptotic gene BCL2 8, 28; Colon cancer stem‐like cell populations, which are highly resistant to 5‐FU treatment, contained high level of miR‐140 and inhibition of miR‐140 activity sensitized these stem‐like cells to 5‐FU treatment by targeting HDAC4 29. MiR‐200 family members have been recently reported to regulate chemoresistance in breast cancer, non‐small cell lung cancer, and pancreatic adenocarcinoma (PDAC), *etc*. through different mechanisms 17, 18, 19. More recently, miR‐425‐5p has been identified as a potential biomarker in renal cell carcinoma, lung squamous cell carcinoma, breast cancer and bladder cancer 20, 22, 30. An up‐regulation of circulating miR‐425‐5p has been observed in head and neck cancer patients after radiotherapy in the blood plasma compared with primary HNSCC (head and neck squamous cell carcinoma) cells 31. It has also been found to be up‐regulated after chemotherapy in oesophageal cancer 32. In addition, miR‐425‐5p has been reported to promote tumorigenicity and aggressiveness in breast cancer and gastric cancer 21, 33. However, the direct involvement of miR‐425‐5p in regulating chemoresistance and the underlying mechanism have not, if at all, been investigated in CRC cells. In our study, we observed that miR‐425‐5p family members were up‐regulated in HCT11‐R cells and inhibition of miR‐425‐5p sensitized these cells to both 5‐FU and OX (Figs 1B and 2). On the contrary, overexpression of miR‐425‐5p conferred resistance of HCT116 cells to these two agents (Fig. 3), demonstrating that miR‐425‐5p plays an important role in regulating chemoresistance in CRC cells. Importantly, co‐administration of miR‐425‐5p inhibitor enhanced chemo response in HCT116‐R xenografts, supporting that miR‐425‐5p can regulate chemoresistance *in vivo* (Fig. 6A–D).

PDCD10 is a recently identified apoptosis‐related gene that has been implicated in mutations associated with cerebral cavernous malformations 34. Previous studies have suggested that the PDCD10 protein may play a role in regulating cell apoptosis. For example, PDCD10 was initially found to be up‐regulated in denervated skeletal muscle atrophy, and overexpression of PDCD10 inhibited natural cell death in fibroblast cell lines exposed to specific apoptosis inducers, such as staurosporine, cycloheximide or tumour necrosis factor‐α 35, 36, suggesting that PDCD10 can function as an anti‐apoptotic protein. However, another study showed that PDCD10 could interact with STK25 to accelerate cell apoptosis under oxidative stress, suggestive of its pro‐apoptotic function 24. These contradictory results indicate that its role in regulating apoptosis maybe cell type and context dependent. Recent microarray data suggested that it may also be involved in tumorigenesis process, since it was reported to be up‐regulated in laryngeal squamous cell carcinoma 37, PDACs 38, metastatic colon cancer cells resistant to cisplatin‐induced apoptosis 39, *etc*. However, the underlying signalling pathways and detailed mechanisms have not been fully elucidated. Using bioinformatics method, combined with experimental approaches, we identified PDCD10 as the directly target of miR‐425‐5p as demonstrated by both Western blot analysis as well as luciferase assay (Fig. 4). Importantly, transient knockdown of PDCD10 eliminated the chemo sensitizing effect of miR‐425‐5p inhibitor, whereas overexpression of PDCD10 recapitulated this effect in HCT116‐R cells (Fig. 5), demonstrating that miR‐425‐5p exerts its function as a regulator of chemoresistance *via* PDCD10 in these CRC cells. Consistently, a reduction in PDCD10 expression by IHC staining was observed in xenografts derived from mice injected with miR‐425‐5p inhibitor compared with control mice (Fig. 6E), supporting that PDCD10 is the functional downstream target of miR‐425‐5p *in vivo*.

In summary, our study demonstrates that miR‐425‐5p is up‐regulated in HCT116‐R cells with acquired resistance to 5‐fluouracil and OX compared with the parental HCT116 cells. Inhibition of miR‐425‐5p increases sensitivity to anti‐cancer drugs by regulating apoptosis‐related protein PDCD10 both *in vitro* and *in vivo*. Therefore, targeting miR‐425‐5p may represent a novel therapeutic approach for CRC patients.

## Conflicts of interest

The authors confirm that there are no conflicts of interest.
